# Dimorphic life cycle through transverse division in burrowing hard coral *Deltocyathoides orientalis*

**DOI:** 10.1038/s41598-022-13347-2

**Published:** 2022-06-07

**Authors:** Asuka Sentoku, Keisuke Shimizu, Tsubasa Naka, Yuki Tokuda

**Affiliations:** 1 Department of Physics and Earth Sciences, University of the Ryukyus, Nishihara, Okinawa 903-0213 Japan; 2 Department of Applied Biological Chemistry, Graduate School of Agricultural and Life Sciences, The University of Tokyo, 1-1-1 Yayoi, Bunkyo, Tokyo 113-8657 Japan; 3 Tottori University of Environmental Studies, 1-1-1 Wakabadaikita, Tottori, 689-1111 Japan

**Keywords:** Animal behaviour, Taxonomy

## Abstract

The azooxanthellate solitary scleractinian *Deltocyathoides orientalis* (family Turbinoliidae), which has bowl-shaped costate corallites, exhibits burrowing behavior on soft substrates and can adapt to an infaunal mode of life. Here, we describe previously unknown aspects of their life history and asexual mode of reproduction based on morphological and molecular phylogenetic analyses. The findings reveal that (1) *D. orientalis* exhibits asexual reproduction by transverse division; (2) smaller bowl-shaped costate anthocyathus derived from cylindrical to tympanoid anthocaulus were attached to hard substrates, including shell fragments and gravels on soft substrates; and (3) anthocyathus only reproduce sexually after division, and anthocaulus was found to regrow and repeatedly produce anthocyathi through transverse division. The bowl-shaped corallum morphology of the anthocyathus just after division might reduce the time required for skeletal formation to enable infaunal adaption after transverse division. Immediately after division, *D. orientalis* can smoothly shift to a burrowing lifestyle that efficiently utilizes soft-substrate environments, thus increasing its survival rate. The morphological formation of prospective anthocyathus in the anthocaulus stage is consequently thought to involve an increase in clonal individuals as well as adaptations for a burrowing free-living mode of life in the anthocyathus stage.

## Introduction

There are more than 1500 known species of scleractinian corals in the phylum Cnidaria, which include zooxanthellate, azooxanthellate, and apozooxanthellate corals^1,2^. Scleractinian corals can exhibit both attached and free-living life modes (e.g., Hoeksema^3^), by attaching to hard substrates such as rocks and reefs, or free-living on soft substrates such as sand and mud. There are two main methods of reproduction in scleractinian corals: sexual and asexual. The asexual reproductive modes include intratentacular budding, extratentacular budding, polyp bail-out, asexual production of embryos, and autotomy in which a part of the body detaches to produce a new individual^4,5^. Furthermore, there are two types of autotomy: transverse division, in which a cut is made perpendicular to the oral-aboral axis of the individual and the individual is divided, and longitudinal division, in which a cut is made parallel to the oral-aboral axis and the body is divided^6^. Although many free-living azooxanthellate corals have been described in the seas around Japan, only a few detailed studies of their populations and ecology have been conducted^7–9^.

Turbinoliids, which are exclusively azooxanthellate and solitary, have a free-living mode of life during their anthocyathus stage (i.e., the upper part of a divided corallum in asexual reproduction)^10^. These corals typically exhibit bowl-shaped, conical, or cylindrical coralla with smaller calicular diameters of less than 1 cm. The family is highly diverse, with 23 extant and 6 fossil genera, and the oldest turbinoliid fossils are collected from the late Cretaceous (Campanian)^10–12^.

The turbinoliid *Deltocyathoides* genus is reserved for those species previously placed in *Peponocyathus* that do not undergo transverse division^10,13^. These corals are free living and bury themselves in soft substrates by transporting substrate sediment upwards along a diagonal path as well as away from the polyp laterally^7^. Sentoku et al*.*^7^ demonstrated that *D. orientalis* has active burrowing and escaping abilities following burial. The infaunal mode of life and the retraction of the oral side of the polyp into the sediment as a reaction to physical stimuli are considered anti-predator strategies, similar to burrowing sea anemones and tube-dwelling anemones. Although little is known about the predators, predation pressure, or predation frequency of this coral, damage repairs have been well recorded in the skeletons of *D. orientalis*. Even highly fragmented individuals preserving less than 10% of their original skeleton are able to regenerate and repair^8^.

*Deltocyathoides orientalis* exhibits slightly flattened bowl-shaped corallites, with an average diameter of 10 mm. There are no juveniles of *D. orientalis* with a calicular diameter of less than 2 mm or a small number of septa^8^. Therefore, little is known about its life cycle especially during its juvenile stage after larvae settlement on soft substrates.

A large number of both living and skeleton samples for two coral species (*D. orientalis* and an undescribed species) were collected during a marine sediment survey off the Pacific and Japanese Sea coasts of Japan. The morphological characteristics of the calice and septal arrangements of these undescribed attached corals is similar to those of free-living *D. orientalis*, but they differ significantly in terms of their corallum forms (cylindrical or tympanoid vs. bowl shape) and modes of life (attached vs. free-living). However, the skeletal specimens of *D. orientalis* collected in this study showed a basal discoloration characteristic of decalcification that occurs in autotomic asexual reproduction (e.g., transverse division^14^).

In this study, morphological and molecular phylogenetic analyses were conducted to elucidate the taxonomic positions of both species and the life cycle and mode of asexual reproduction of *D. orientalis*.

## Results

### Corallum morphology

#### Deltocyathoides orientalis (Anthocyathus)

**Figure 1:**
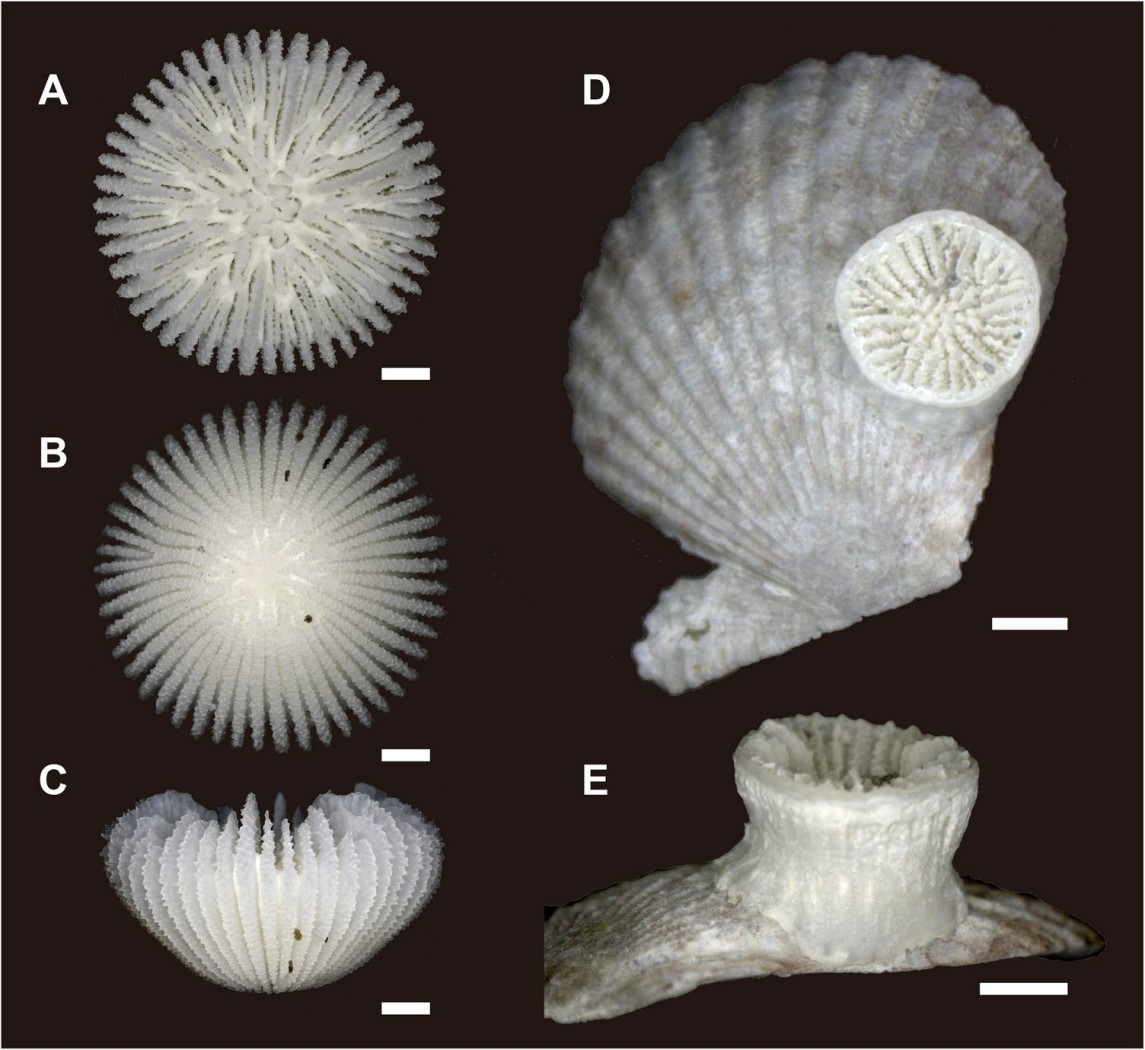
(**A**–**C**) Oral (top), bottom, and lateral views of *Deltocyathoides orientalis*. (**D**, **E**) oral (top) and lateral views *of* undescribed specie. Scale bars = 1 mm.

**Figure 2:**
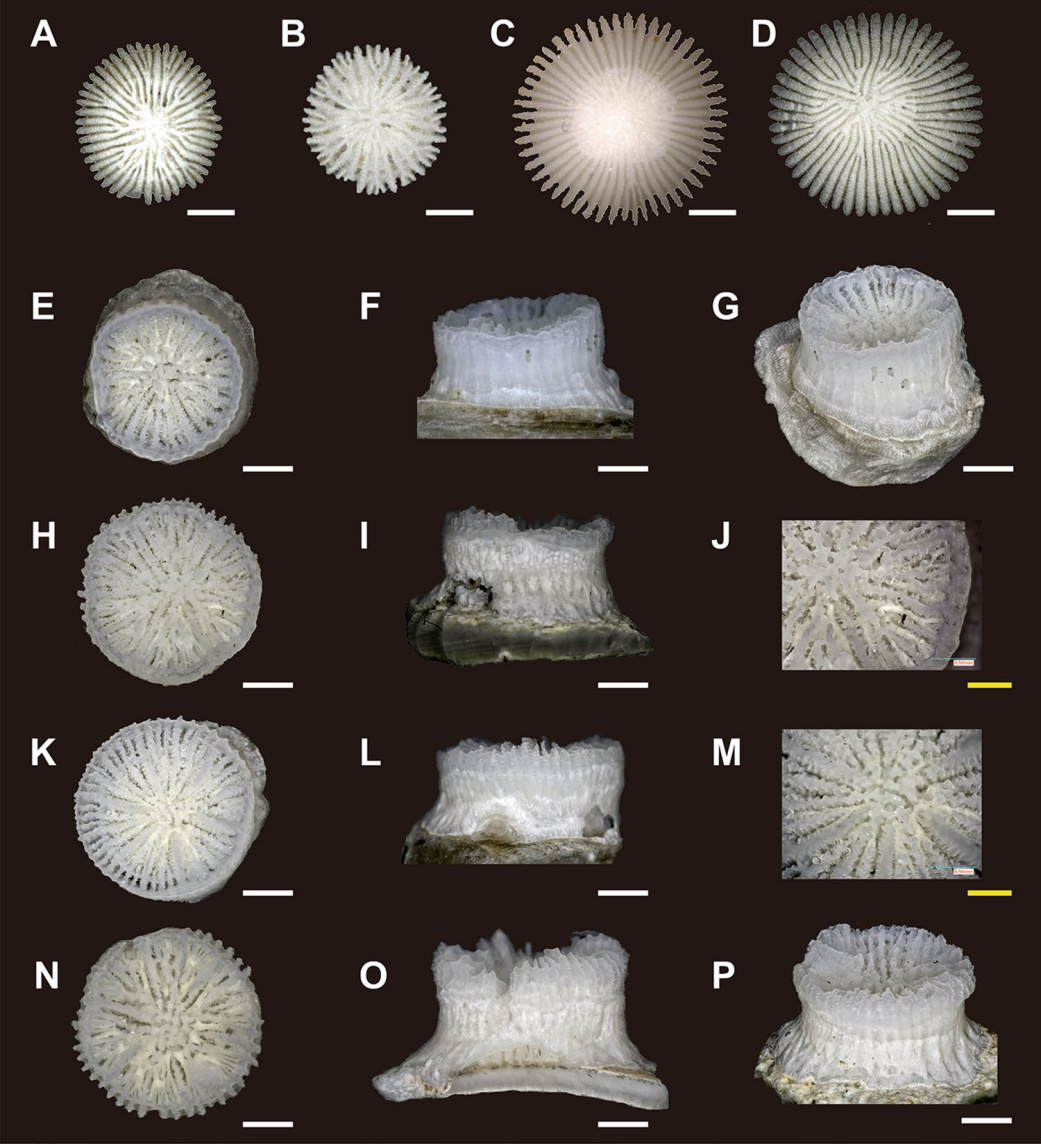
(**A**–**D**) Anthocyathi of *Deltocyathoides orientalis* with decalcification process scar. (**E**–**P**) oral (top), lateral, and other views *of* undescribed species (anthocauli of *Deltocyathoides orientalis*). (**E**–**G**) 501*.* (**H**–**J**) 503*.* (**K**–**M**) 505*.* (**N**–**P**) 507*.* White scale bars = 1 mm, Yellow scale bars = 0.5 mm.

**Figure 3:**
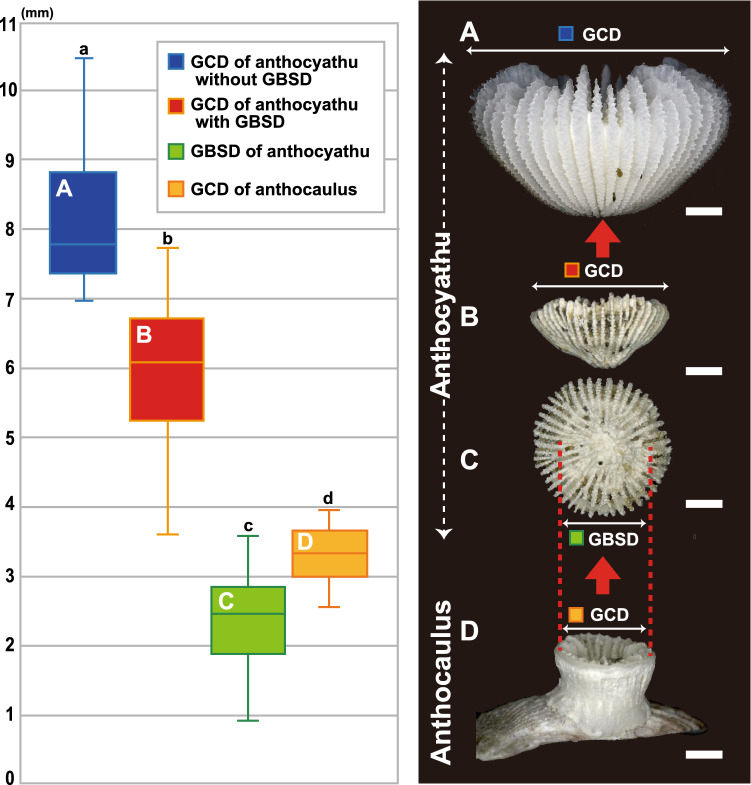
Box plots indicating the size distributions for the GCD of anthocyathi, GBSD, and GCD of the anthocauli for four abundance quartiles, and the photo characteristics measured for each specimen. The data are based on 41 specimens of anthocyathi and 11 specimens of anthocauli. The center line of each box plot represents the median abundance; the lower and upper edges of the boxes denote the 25th and 75th percentiles, respectively; and the whiskers denote the maximum and minimum percentiles. (**A**) Lateral views of GCD of anthocyathus without GBSD*.* (**B**) Lateral view of the GCD of anthocyathus with GBSD. (**C**) Bottom view of the GBSD of anthocyathus. (**D**) Lateral views of the GCD of anthocaulus (undescribed species). Scale bars = 1 mm.

Ten *D. orientalis* (Anthocyathus) individuals were collected from Station (St.) 1 and 308 individuals from St. 2. *D. orientalis* inhabits soft-bottom substrates and possesses a conical or bowl-shaped skeleton completely covered by soft tissue (Figs. 1A–C, 2A–D). The mouth is in the center of the upper part of the corallum (calice) and is encircled by 48 tentacles. The greater calicular diameter (GCD) and lesser calicular diameter (LCD) of *D. orientalis* without greater basal scar diameter (GBSD) are 7.03–10.58 mm and 6.72–10.57 mm (Fig. 3A), respectively, and the height of the corallum (HT) is up to 5.20 mm. At the thecal lateral faces the costae are ridged, granular, very prominent, and separated by deep intercostal grooves. At the base, intercostal grooves are shallow. Higher cycles of costae (third and fourth) originate from bi- or trifurcations. A circular decalcification scar was observed on the base of the skeleton of 41/308 *D. orientalis* specimens collected at St. 2 (Fig. 2A–D). The intercostal in the center of the decalcification scar were indistinct. The center of the basal part of the smaller corallum is comprised of protuberances and encircled by costae. Within the corallum, the costal edge angle is bimodal, and the angle increases (approximately 20–40°) at the periphery of the decalcified part. The GCD and LCD of *D. orientalis* with GBSD are 3.61–7.75 mm and 3.34–7.66 mm, respectively (Fig. 3B). The GBSD and lesser basal scar diameter (LBSD) are 0.91–3.59 mm and 0.91–3.29 mm (Fig. 3C), respectively.

The septa were ordinarily hexamerally arranged in four complete cycles according to the following formula: S1 > S2 > S4 > S3 (48 septa; Sx, cycle of septa). Sublamellar to styliform pali exist before all but the last cycle of septa (P1–3; Px, cycle of pali). The columella rudimentary is composed of a few granulated and interconnected pillars. S1 are independent and almost reach the columella and bear a small pali. The inner septal edges of S1 are slightly sinuous. The P1 are usually indistinguishable from the columellar elements. S2 are approximately 3/4 the width of S1. P2 are three times wider than P1. S3 are approximately 1/2 the size of S2. The thinnest and most recessed P3 are present. Axial edge of P3 fuses to the distal edge of adjacent P2. The S4 are dimorphic in size: those adjacent to S1 are wider than S3 and those adjacent to S2 are approximately as wide as S3. Axial edge of each S4 fuses to distal edge of P3.

#### Undescribed species (Anthocaulus of *Deltocyathoides orientalis*)

**Figure 4:**
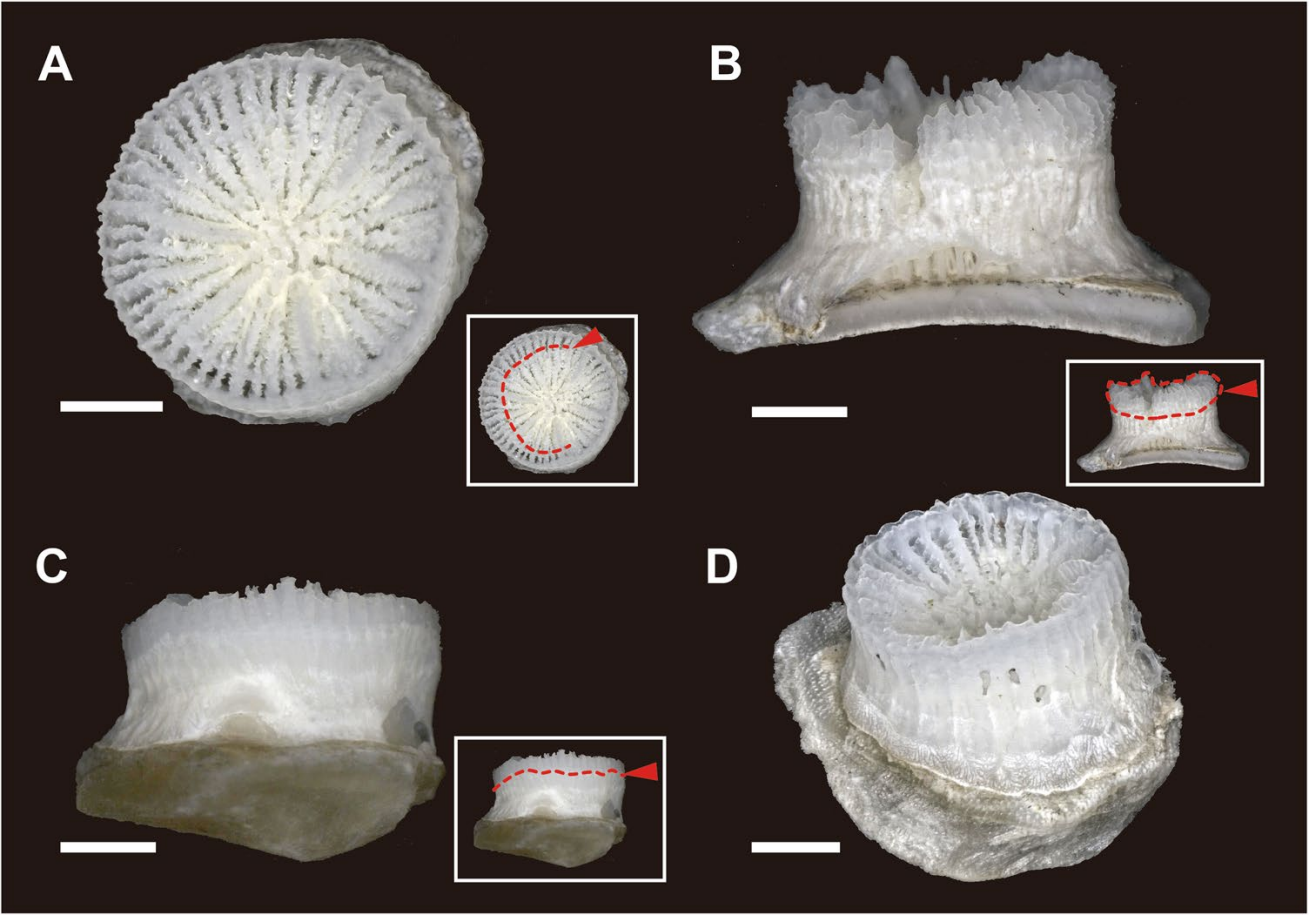
Undescribed species (anthocauli of *Deltocyathoides orientalis)* with transverse division scar. Red dotted lines and arrows indicate the auxiliary lines of the division scar. (**A**) Circular walls formed inside the calice as in a marked rejuvenescence. (**B**) primary septa protrude above corallite, and upper part of corallite is bulging. (**C**) Decalcification scar developed perpendicular to the growth direction of the corallite. (**D**) The center of the calice is depressed, and the upper part of the septum is curved toward the center. Scale bars = 1 mm.

Four individuals of an undescribed species were collected from St. 1 and seven individuals from St. 2 (Fig. 2E–P). These specimens exhibited cylindrical to tympanoid corallum morphology and were attached to shell fragments. The GCD, LCD, and HT were 2.56–3.95 mm (Fig. 3D), 2.36–3.90 mm, and 1.38–2.64 mm, respectively. The calice was encircled by a thin wall with faint costae of granular ridges on its surface. Two or more white opaque lines on the outside of the wall appeared perpendicular to the growth direction of the corallum (Figs. 2L,O, 4C). Some specimens showed exerted septa (especially in S1) and distinct costae only in the uppermost part of the corallum (Fig. 4B). In contrast, some specimens exhibited a depressed center of calice with sloped septal edges toward its center without the exerted septa and costae (Fig. 4D). Another wall was occasionally formed within the calice, similar to a marked rejuvenescence (Fig. 4A). The septa were hexamerally arranged in four complete cycles according to the following formula: S1 > S2 > S4 > S3 (48 septa; except for specimen number 501 which exhibited 38 septa). Columella rudimentary were composed of few granulated and interconnected pillars. S1 was the only independent septa, almost reaching the columella with a slightly sinuous axial edge, bearing small palus usually indistinguishable from the columellar elements. S2 is approximately 3/4 the width of S1. S3 is approximately 1/2 the size of S2, bearing the thinnest. S4 is dimorphic: those adjacent to S1 were wider than S3 and those adjacent to S2 were approximately as wide as S3. Axial edge of each S4 fuses to distal edge of S3.

### Morphological comparison between anthocaulus and anthocyathus

**Figure 5:**
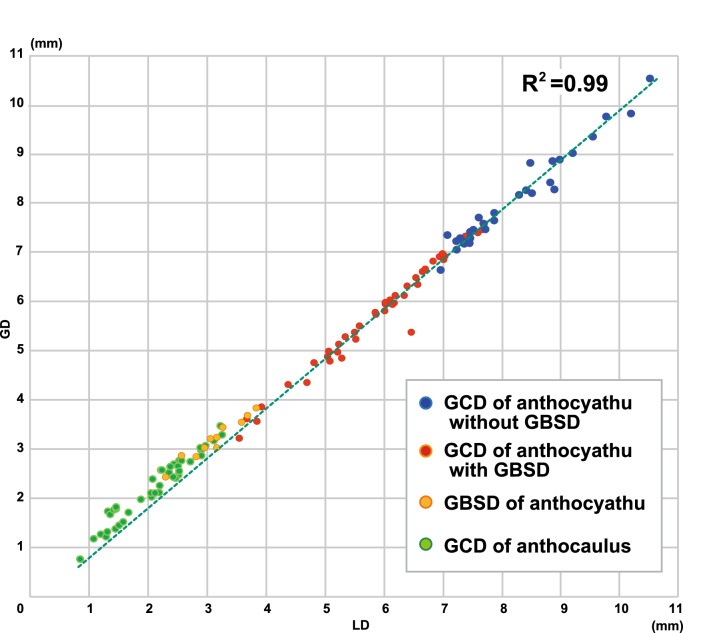
Relationship between the GCD/LCD of Anthocyathu without GBSD, GCD/LCD of Anthocyathu with GBSD, GBSD/LBSD of Anthocyathu and GCD/LCD of Anthocaulus (R2 = 0.99, N = 125).

The GCD with the GBSD (n = 42), GBSD (n = 42) of the anthocyathi, GCD without GBSD of the anthocyathi (n = 30), and GCD of the anthocauli (n = 11) were measured to compare their size distributions. The size distributions of the GBSD (mean = 2.35 mm, σ = 0.63), GCD with GBSD (mean = 5.95 mm, σ = 1.07), and the GCD without GBSD (mean = 8.23 mm, σ = 0.96) of the anthocyathi were found to be significantly different (Steel–Dwass's multiple comparison test; p < 0.001, Supplementary Table S1). The GBSD of the anthocyathi is slightly smaller than the GCD of anthocauli (mean = 3.32 mm, σ = 0.39; Fig. 3, Steel–Dwass’s multiple comparison test; p < 0.001, Supplementary Table S1). The relationship between the GCD/LCD with GBSD, GBSD/LBSD, and GCD/LCD without GBSD of Anthocyathi and GCD/LCD of Anthocauli is shown in Fig. 5. The ratios of the greater and lesser diameters at the four measurement points were strongly positively correlated (R^2^ = 0.99, n = 125). The size distributions of the GCD of anthocyathi without GBSD (p = 0.26), GCD of anthocyathi with GBSD (p = 0.83), GBSD of anthocyathi (p = 0.77), and GCD of anthocauli (p = 0.99) approximated a normal distribution, according to the Kolmogorov–Smirnov test (Supplementary Table S2).

### Molecular phylogenetic analyses

**Figure 6:**
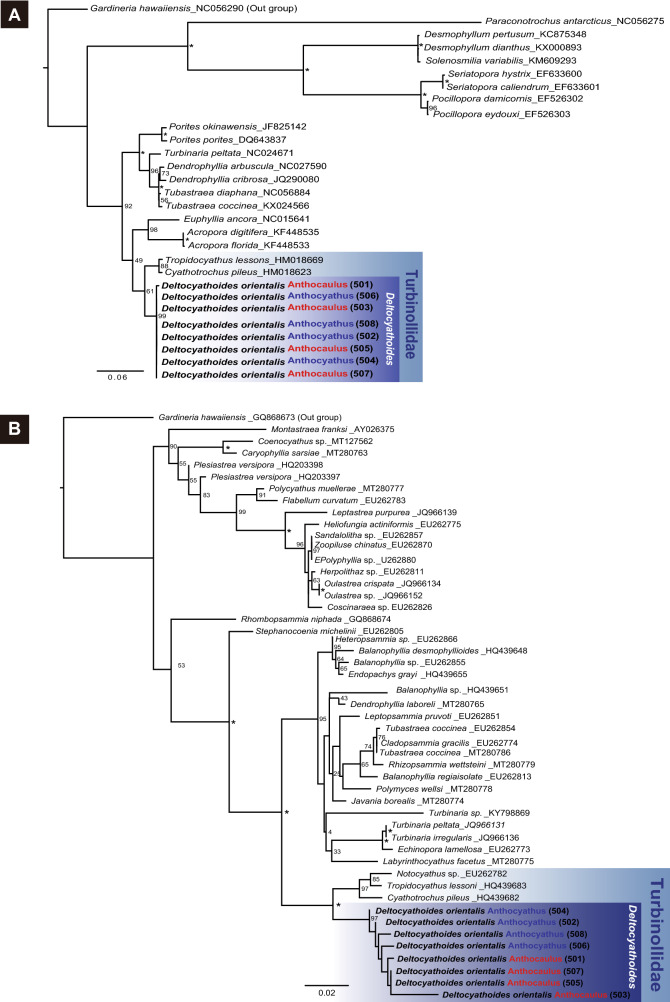
Phylogenetic analyses of the scleractinian corals based on two regions the mitochondrial COI (**A**) and the nuclear 28S ribosomal DNA (**B**) regions. All maximum likelihood trees were constructed using RA×ML with the GTRCAT model and 1000 bootstrap replicates. Numbers on the nodes indicate the bootstrap values. Asterisks indicate 100% bootstrap support.

Phylogenetic analysis was carried out using the maximum likelihood (ML) method, and it placed *D. orientalis* and the other undescribed species into a single clade in five regions (mitochondrial 12S ribosomal DNA (Supplementary Fig. S1A), 16S ribosomal DNA (Supplementary Fig. S1B), and cytochrome c oxidase subunit I (COI) (Fig. 6A) regions and nuclear 28S ribosomal DNA (Fig. 6B) and internal transcribed spacer (ITS) (Supplementary Fig. S1C)).

Turbinoliidae, *D. orientalis,* and other undescribed species were placed in a single clade by four regions other than ITS. Sequence data for the ITS region of Turbinoliidae were not available in previous studies. For the 16S ribosomal DNA (462 bp) and CO1 (646 bp), the sequences of all eight samples of *D. orientalis* and the undescribed species were identical. For the mitochondrial 12S ribosomal DNA region, sequence data from six samples were used, as the sequences of two undescribed species (specimen numbers 501 and 505) were unavailable. There was one variable site among the 906 bp 12S ribosomal DNA sequences. For the nuclear 28S ribosomal DNA region of the eight samples, there were 11 variable sites (1.58%) among the 698 bp. For the ITS region of the eight samples, there were 19 variable sites (3.51%) among the 541 bp.

## Discussion

### Transverse division of *Deltocyathoides orientalis*

In this study, we conducted a detailed morphological analysis of an undescribed species and *D. orientalis*. The results showed that the two species were different in terms of their mode of life (attached vs. free-living) and GCD size (Figs. 3 and 5, Supplementary Table S3). However, some morphological characteristics such as granular septa, septal and palar arrangement, and columella were common between the two species.

A circular discoloration was observed on the basal part of the skeleton in 41/308 specimens of free-living *D. orientalis* collected at St. 2 (Fig. 2A–D). Similar discoloration by decalcification for transverse division has previously been observed at the basal part of almost all anthocyathi of *Truncatoflabellum*^14,15^. However, over 80% of the specimens of free-living *D. orientalis* did not show this basal discoloration (Fig. 5). Soft tissues on the basal part of the anthocyathus of *Truncatoflabellum spheniscus* are immediately lost after transverse division^14^. Consequently, the original basal scar and the decalcified skeleton remained without additional skeletal thickening. As the *D. orientalis* has soft parts covering the entire skeleton when it is alive, the outer surface of the corallum, including the discolored basal part, also undergoes additional thickening of the associated skeletal region. This means that the basal parts of the corallum of *D. orientalis* may not all exhibit discoloration that is hidden under the new intact skeletons. This consideration corresponds to the smaller size distribution of the GCD of anthocyathus bearing basal scars in *D. orientalis*, which is thought to have a shorter duration of skeletal precipitation than the GCD of anthocyathus without it (Fig. 3, Supplementary Table S1). In addition, Sentoku et al*.*^8^ showed that *D. orientalis* is capable of self-repair if 10% of the skeleton remains after physical damage, in which the basal discoloration can potentially be lost. Therefore, discoloration restricted to the basal part of *D. orientalis* is inferred to be generated by decalcification, and almost all *D. orientalis* may inherently have a discolored basal part.

In the attached specimens, white opaque discoloration lines on the outside of the wall perpendicular to the growth direction and double-walled structures, such as rejuvenescences, were observed (Fig. 4A–C). Anthocauli of Pliocene fossils of *Truncatoflabellum carinatum* and extant *Truncatoflabellum* spp. show successive rejuvenescences that are derived from a temporal decrease in polyp diameter due to transverse division injury^14,15^. Moreover, the anthocauli of extant *Truncatoflabellum* spp. exhibit traces of decalcification at the uppermost periphery of the outer wall portion of the rejuvenescence. A similar trace of decalcification of anthocaulus has been reported in *Fungia fungites*^16^. The discoloration and double-walled structures in the attached specimens are thought to have been formed by transverse division with decalcification.

Furthermore, the nucleotide sequences of the undescribed species and *D. orientalis* were completely consistent in the 16S-rDNA region (462 bp) and CO1 region (646 bp). In addition, due to the creation of phylogenetic trees using the nucleotide sequences obtained from the five gene regions, the undescribed species and *D. orientalis* were found to represent a single clade in all gene regions (Fig. 6A–B, Supplementary Fig. S1A–C).

Based on the results of both the morphological and molecular phylogenetic analyses, we concluded that the undescribed species was the anthocaulus of *D. orientalis*. Moreover, the free-living anthocyathus of *D. orientalis* reproduces asexually by transverse division of the attached anthocaulus.

### Control of anthocyathus morphology by transverse division

Two calical morphologies were observed in the attached coralla: the depressed calice and the exerted and bulged calice with distinct costae (Fig. 4). Particularly, the skeletal characteristics of the uppermost part of the latter attached specimens were found to closely resemble those of the anthocyathus of *D. orientalis* (Fig. 4B). The smaller corallum of the anthocyathus of *D. orientalis* shows a slightly protruding base with decalcification. The center of the basal part is composed of tubercular fragments from the divided columella of the anthocaulus and is encircled by costae. From the side view, the costal edge angle is bimodal, and the angle increases (from approximately 20°–40°) at the periphery of the decalcification part. The protruding morphology of the lower section of the anthocyathus of *D. orientalis* fits into the depressed calice morphology of the anthocauli. Thus, the exerted and bulged calical part with the deeper part of the inner calice of the anthocaulus without the outer wall of *D. orientalis* corresponding to slightly bowl-shaped anthocyathus may be scooped from anthocaulus by transverse division. Since only the inside of the calice was extracted from the anthocaulus, the GBSD of the anthocyathus of *D. orientalis* was only slightly smaller than the GCD of anthocaulus (Fig. 3).

The bowl-shaped corallum and distinct costae of *D. orientalis* plays an important role when burrowing into soft substrates^7,8^. The corallum morphology of the anthocyathus with a protruding base immediately after division might have an ecological function that facilitates free living on soft substrates. The anthocyathi of *Truncatoflabellum* show truncated basal scars that are almost horizontal or along the arcuate growth line of the wall surface^6,14^. In addition, the increased thickening deposits in the lower parts of the anthocyathus of *Truncatoflabellum* might contribute towards increased stability in the substrate, as well as maintaining the calice orientation towards the sea surface, which would be advantageous for the purposes of food acquisition^14^. Thus, the morphological formation patterns of the prospective anthocyathus by skeletal growth and carving by decalcification in the anthocaulus stage are thought to involve not only increasing clonal individuals but also adaptations for a free-living mode of life after transverse division in the anthocyathus stage.

### Alternation of generations by transverse division

Statistical examination with Steel–Dwass's multiple comparison test showed that the size distributions of GCD and GBSD for the anthocyathi in *D*. *orientalis* were distinctly different (Fig. 3, Supplementary Table S1). The size distribution of the basal scars approximates a normal distribution (Supplementary Table S2), suggesting that transverse divisions only occur in coralla that reach a certain diameter, not randomly. The size distributions of the GCD of the anthocyathi are significantly different from GBSD (Supplementary Table S1). The difference indicates that the anthocyathus of *D*. *orientalis* does not show asexual reproduction by transverse division. In contrast, plural white opaque horizontal lines of decalcification on the outside of the wall of the anthocauli of D. *orientalis* indicate repeated transverse divisions (Figs. 2L,O, 5C). The statistical analysis supports the idea that the anthocauli of D. *orientalis* repeatedly reproduce asexually through transverse division, whereas the anthocyathi only reproduce sexually. *D. orientalis*, thus, exhibits a distinct alternation of generations (sexual in anthocyathi vs. asexual in anthocauli), which is also well known in *Fungia* and *Truncatoflabellum*^6,14,15,17^. Differences were observed in the number of anthocauli (seven individuals) and anthocyathi (384 individuals) of *D. orientalis* collected at St. 2. The small number of the anthocauli of D. *orientalis* is similar to the anthocauli of *Truncatoflabellum*^15^. These larger increasing rates of anthocyathus suggest that asexual reproduction may play an important role in increasing coral population size in soft-substrate environments, such as the sand and mud.

**Figure 7:**
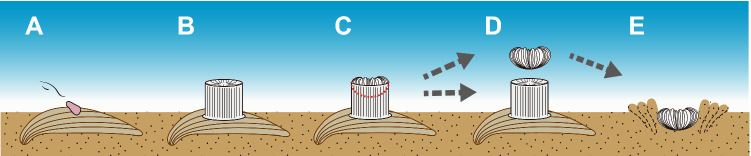
Schematic diagram showing the dimorphic life cycle of the azooxanthellate scleractinian coral *Deltocyathoides orientalis*. (**A**) Coral planula attaching to shell fragment on soft substrate. (**B**) Anthocaulus. (**C**) Anthocyathus occurring at the upper interior of the anthocaulus. (**D**) Division of anthocyathu from anthocaulus. (**E**) Anthocyathus burrowing into a soft-bottom substrate immediately after division.

Anthocyathi of *Peponocyathus duncani*^18^ and *Bourneotrochus stellulatus*^19^ show repeated asexual reproduction by transverse division (e.g.^20–22^). Zibrowius^23^ and Stolarski^21^ discussed importance of the transverse divisions of anthocyathi for adaptive strategies on soft substrates, as well as the increase in clonal individuals^21,23^. Corallum size and weight reduction by the transverse division of the anthocyathus could lead to efficient automobility in *Peponocyathus duncani* having a smaller cylindrical corallum with a maximum calicular diameter of 3.7 mm^21^. However, the morphological analysis of *D. orientalis* revealed that the anthocyathi did not undergo transverse division. The acquisition of automobility in anthocyathi of *D. orientalis*, including burrowing, escape from burial, and righting behaviors, implies that the species is actively utilizing habitats under the seafloor. The ability of *D. orientalis* to retract the oral side of the polyp into the sediment is considered an anti-predator response, similar to that of burrowing sea anemones and tube-dwelling anemones^7^. The asexual reproduction of division that occurs after the formation of a bowl-shaped corallum with costae in the anthocaulus stage suggested in this study reduces the cost and time of skeletal formation for infaunal adaptation after transverse division. Immediately after division, *D. orientalis* can smoothly shift to a burrowing lifestyle that efficiently utilizes the soft-substrate environments, which probably increases its survival rate (Fig. 7).

In this study, we clarified the life history of *D. orientalis* based on morphological and molecular phylogenetic analyses. In the future, it will be necessary to clarify the frequency of sexual and asexual reproduction and the detailed growth pattern of the species by observing the gonadal development of anthocyathi and the ecology of anthocauli. Furthermore, since *Deltocyathoides* has been classified based on the presence or absence of transverse division with the closely related genus *Peponocyathus*, it will also be necessary to systematically revise both genera in the future.

## Methods

### Specimens

Samples were collected off the Pacific coast of Ohakozaki, Japan (39° 21.966110 N, 142° 01.59220 E; St.1), at depths of 104 m, and Hachinohe, Japan (40° 30.08620 N, 141° 50.85440 E; St.2), at depths of 195 m. We examined 318 individuals of *D. orientalis* and 11 individuals of an undescribed species of solitary coral attached to shells (Fig. 1). Of these, four individuals with soft parts were selected for molecular phylogenetic analyses (Fig. 2). Additionally, individuals with basal scars formed at the bottom of *D. orientalis* were also selected for morphological analysis. Details of the macro-skeletal features were observed using a stereomicroscope (Leica M165C) and digital microscope (Keyence VHX-7000). The relevant corallae were photographed at various angles and magnifications for documentation purposes. Where necessary, measurements were performed using Adobe Photoshop (Adobe Inc., CA, USA), ImageJ (NIH, MA, USA)^24^, and electronic calipers. These data were statistically verified using Steel–Dwass’s multiple comparison test. The size distributions of the GCD and GBSD were assessed for the goodness of fit of the normal distributions using the Kolmogorov–Smirnov test (Supplementary Table S2).

### DNA preparation, amplification, and sequence analyses

Four *D. orientalis* with soft parts (specimen numbers: 502, 504, 506, and 508) and four undescribed species (specimen numbers: 501, 503, 505, and 507) were selected for DNA extraction. Specimens of the entire system (including the skeleton) were extracted and immersed in a lysis buffer. Genomic DNA was extracted using a DNeasy Tissue Kit (QIAGEN, Hilden, Germany). DNA concentrations were determined using a Nanodrop 1000 (Thermo Scientific) prior to Polymerase Chain Reaction (PCR) amplification under the following conditions:

1. 16S rDNA, the primers developed by Le Goff-Vitry et al*.*^25^ (LP16SF 5′-TTGACCGGTATGAATGGTGT and LP16SR 5′-TCCCCAGGGTAACTTTTATC) were used to amplify a fragment of 462 bp.
2. COX1, the universal primers developed by Folmer et al*.*^26^ (LCO1 490 5′-GGTCAACAAATCATAAAGATATTGG and HCO2 198 5′-TAAACTTCAGGGTGACCAAAAAATCA) were used to amplify a fragment of 646 bp.
3. For 12S rDNA, the primers developed by Chen and Yu^27^ ANTMT12SF 5′-AGCCACACTTTCACTGAAACAAGG and ANTMT12SR 5′-GTTCCCYYWCYCTYACYATGTTACGAC) were used to amplify a fragment of approximately 905 bp.
4. 28S rDNA primers developed by Medina et al*.*^28^ (28S.F63sq 5′-AATAAGCGGAGGAAAAGAAAC and 28S.R635sq 5′-GGTCCGTGTTTCAAGACGG) was used to amplify a fragment of approximately 700 bp.
5. ITS1-5.8rRNA-ITS2—the primers developed by McFadden^29^ (1S-GGTACCCTTTGTACACACCGCCCGTCGCT and 2SS-GCTTTGGGCGGCAGTCCCAAGCAACCCGACTC) were used to amplify a fragment of approximately 540 bp.

PCR cycling conditions were as follows: 95 °C for 4 min, followed by four cycles of 30 s at 94 °C, 60 s at 50 °C, 120 s at 72 °C, and 30 cycles of 30 s at 94 °C, 60 s at 55 °C, 120 s at 72 °C, and finally 4 min at 72 °C. The PCR products were run on 1.5% agarose gel, extracted from the gel, and purified using the Wizard SV Gel and PCR Clean-Up System (Promega, Madison, WI, USA). The fragments were directly sequenced on a 3130 Genetic Analyzer (Applied Biosystems, Foster City, CA, USA) with a BigDye Terminator v1.1 or v3.1 Cycle Sequence Kit (Applied Biosystems). The sequences were deposited in GenBank (Accession numbers, see Supplementary Table S3).

Six to eight sequences from the five regions gathered in this study were combined with 137 sequences derived from previous studies (Supplementary Fig. S1, Supplementary Table S3). The sequences were aligned using the online version of MAFFT (v7.503; http://mafft.cbrc.jp/alignment/server/, last accessed February 2nd, 2022^30,31^). The gap regions were trimmed using TrimAl (1.2rev59)^32^. Maximum likelihood trees were constructed using RaxML^33^ under the GTRCAT model^34^ with 1000 bootstrap replications. The sequences of *Gardineria hawaiiensis* were used as outgroups, except for the ITS region analysis. All phylogenetic trees were visualized and edited using FigTree v1.4.4 (http://tree.bio.ed.ac.uk/software/figtree/, last accessed February 2nd, 2022).

## Supplementary Information

Supplementary Legends.

Supplementary Figure S1.

Supplementary Figure S1.

Supplementary Figure S1.

Supplementary Table S1.

Supplementary Table S2.

Supplementary Table S3.

## Data Availability

The data underlying this article are available in the GenBank/EMBL/DDBJ database at https://www.ddbj.nig.ac.jp/index-e.html, and can be accessed with accession numbers LC685944-LC685965 and LC686129-LC686144.

## References

[1] Roberts JM, Wheeler A, Freiwald A, Cairns SD (2009). Cold-Water Corals: The Biology and Geology of Deep-Sea Coral Habitats.

[2] Madin JS (2016). The Coral Trait Database, a curated database of trait information for coral species from the global oceans. Sci. Data..

[3] Hoeksema BW (1993). Phenotypic corallum variability in Recent mobile reef corals. Courier Forschungs-Institut Senckenberg.

[4] Serrano E, Coma R, Inostroza K, Serrano O (2017). Polyp bail-out by the coral *Astroides calycularis* (Scleractinia, Dendrophylliidae). Mar. Biodivers..

[5] Goffredo S (2012). Unusual pattern of embryogenesis of *Caryophyllia inornata* (Scleractinia, Caryophylliidae) in the Mediterranean Sea: Maybe agamic reproduction?. J. Morphol..

[6] Cairns, S. D. Asexual reproduction in solitary Scleractinia. in *Proc. 6th Int. Coral Reefs Cong*. **2**, 641–646 (1988).

[7] Sentoku A, Tokuda Y, Ezaki Y (2016). Burrowing hard corals occurring on the sea floor since 80 million years ago. Sci. Rep..

[8] Sentoku A, Tokuda Y, Ezaki Y, Webb GE (2018). Modes of regeneration and adaptation to soft-bottom substrates of the free-living solitary scleractinian *Deltocyathoides orientalis*. Lethaia.

[9] Sentoku A, Tokuda Y (2021). New records of azooxanthellate scleractinian corals (Cnidaria: Anthozoa) from Sagami Bay and Suruga Bay, Japan. Zool. Sci..

[10] Cairns SD (1997). A generic revision and phylogenetic analysis of the Turbinoliidae (Cnidaria: Scleractinia). Smithson. Contrib. Zool..

[11] Filkorn HF (1994). Fossil scleractinian corals from James Ross basin, Antartica. Antarct. Res. Ser..

[12] Cairns SD (2004). The azooxanthellate Scleractinia (Coelenterata: Anthozoa) of Australia. Rec. Aust. Mus..

[13] Kitahara MV, Cairns SD (2021). *Azooxanthellate scleractinia* (Cnidaria, Anthozoa) from New Caledonia. Mem. Museum Natl. Hist. Nat..

[14] Tokuda Y, Haraguchi H, Ezaki Y (2017). First real-time observation of transverse division in azooxanthellate scleractinian corals. Sci. Rep..

[15] Tokuda Y, Ezaki Y (2012). Asexual reproduction of Pliocene solitary scleractinian coral truncatoflabellum: A morphological and biometric study. J. Paleontol..

[16] Linnaeus, C. Systema Naturae per regna tria naturae, secundum classes, ordines, genera, species, cum characteribus, differentiis, synonymis, locis. *Editio decima, reformata* (10th revised edition), 1, 824 (1758).

[17] Yamashiro H, Yamazato K (1987). Studies on the detachment of the discs of the mushroom coral Fungia Fungites with special reference to hard structural changes. Galaxea.

[18] Reuss AE (1871). Die fossilen Korallen des österreichisch-ungarischen Miocäns. Denkschriften der kaiserlichen Akademie der Wissenschaften. Math. Naturwiss. Cl..

[19] Cairns SD (1984). New records of ahermatypic corals (Scleractinia) from the Hawaiian and Line Islands. Bish. Mus. Occas. Pap..

[20] Wells JW (1984). Notes on Indo-Pacific scleractinian corals. Part 10. Late Pleistocene ahermatypic corals from Vanuatu. Pac. Sci..

[21] Stolarski J (1992). Transverse division in a Miocene scleractinian coral. Acta Palaeontol. Pol..

[22] Cairns SD (1995). The marine fauna of New Zealand: Scleractinia (Cnidaria: Anthozoa). N. Z. Oceanogr. Mem..

[23] Zibrowius, H. Asexual reproduction by bud-shedding in shallow-water Balanophylla of the tropical Indo-Pacific (Cnidaria: Scleractinia: Dendrophylliidae). in *Proc. 5th Int. Coral Reefs Cong*. **5**, 233–238 (1985).

[24] Schneider CA, Rasband WS, Eliceiri KW (2012). NIH Image to ImageJ: 25 years of image analysis. Nat. Methods.

[25] Le Goff-Vitry MC, Rogers AD, Baglow D (2004). A deep-sea slant on the molecular phylogeny of the Scleractinia. Mol. Phylogenet. Evol..

[26] Folmer O, Black M, Hoeh W, Lutz R, Vrijenhoek R (1994). DNA primers for amplification of mitochondrial cytochrome c oxidase subunit I from diverse metazoan invertebrates. Mol. Mar. Biol. Biotechnol..

[27] Chen CA, Yu JK (2000). Universal primers for amplification of mitochondrial small subunit ribosomal RNA-encoding gene in scleractinian corals. Mar. Biotechnol. (NY).

[28] Medina M, Collins AG, Takaoka TL, Kuehl JV, Boore JL (2006). Naked corals: Skeleton loss in Scleractinia. Proc. Natl Acad. Sci. USA.

[29] McFadden D (2001). Economic choices. Am. Econ. Rev..

[30] Katoh K, Rozewicki J, Yamada KD (2019). MAFFT online service: Multiple sequence alignment, interactive sequence choice and visualization. Brief. Bioinform..

[31] Kuraku S, Zmasek CM, Nishimura O, Katoh K (2013). Leaves facilitates on-demand exploration of metazoan gene family trees on MAFFT sequence alignment server with enhanced interactivity. Nucleic Acids Res..

[32] Capella-Gutiérrez S, Silla-Martínez JM, Gabaldón T (2009). trimAl: A tool for automated alignment trimming in large-scale phylogenetic analyses. Bioinformatics.

[33] Stamatakis A (2014). RAxML version 8: A tool for phylogenetic analysis and post-analysis of large phylogenies. Bioinformatics.

[34] Stamatakis, A. Phylogenetic models of rate heterogeneity: A high performance computing perspective. in *Proc. IPDPS2006, Rhodos, Greece* (2006).

