# Supplementary material for: Dimorphic life cycle through transverse division in burrowing hard coral Deltocyathoides orientalis
Source: Sci Rep. 2022 Jun 7;12:9359. doi: 10.1038/s41598-022-13347-2 (PMC9174229; doi:10.1038/s41598-022-13347-2)
Supplement: Supplementary file 1 — Supplementary Legends. [file 41598_2022_13347_MOESM1_ESM.docx]

**Dimorphic life cycle through transverse division in the burrowing hard coral *Deltocyathoides orientalis***

**Asuka Sentoku^1^*, Keisuke Shimizu^2^, Tsubasa Naka^1^ and Yuki Tokuda^3^**

^1^Department of Physics and Earth Sciences, University of the Ryukyus, Nishihara,

Okinawa 903-0213, Japan

^2^Department of Applied Biological Chemistry, Graduate School of Agricultural and Life Sciences, The University of Tokyo, 1-1-1 Yayoi, Bunkyo, Tokyo, 113-8657, Japan.

^3^Tottori University of Environmental Studies, 1-1-1 Wakabadaikita, Tottori 689-1111, Japan.

**Supplementary information**

Supplementary Table S1. GenBank accession numbers of the genes used in the phylogenetic analyses.

Supplementary Table S2. Steel-Dwass multiple comparison test results

Supplementary Figure S1. Phylogenetic analyses results for the scleractinian corals based on three regions of the mitochondrial 12S ribosomal DNA (A), 16S ribosomal DNA (B), and ITS (C) regions. All maximum likelihood trees were constructed using RAxML under GTRCAT model with 1000 bootstrap replicates. Numbers on the nodes indicate the bootstrap values. Asterisks indicate 100% bootstrap support.
